# Supplementary material for: APOBEC SBS13 Mutational Signature—A Novel Predictor of Radioactive Iodine Refractory Papillary Thyroid Carcinoma
Source: Cancers (Basel). 2022 Mar 21;14(6):1584. doi: 10.3390/cancers14061584 (PMC8946015; doi:10.3390/cancers14061584)
Supplement: Supplementary file 1 [file cancers-14-01584-s001.zip › SupplementaryFigures.pdf]

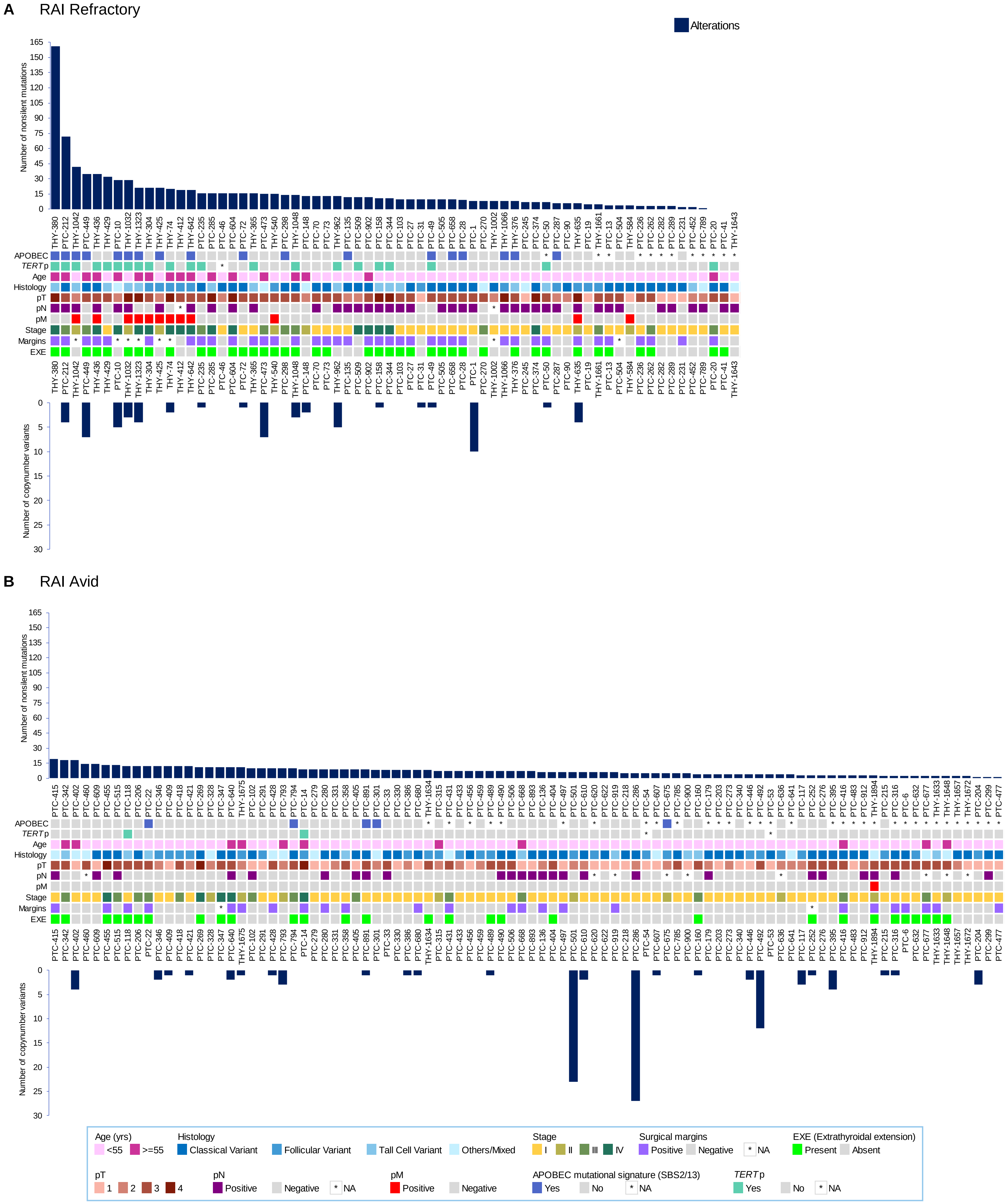

Figure S1: Genomic landscape of RAI (A) refractory (n=66) and (B) avid (n=92) PTC cohort (total n=158). The total number of nonsilent mutations (top panel) and copy number variants, including gains, losses, amplifications and deletions (bottom panel) detected in each patient, with clinical details coded below. *TERT* promoter mutation (C228T and C250T) status was acquired via additional Sanger Sequencing. Extra-thyroidal extension (EXE) includes both microscopic (tumor extending beyond thyroid capsule, without visual evidence) and macroscopic EXE (visually evident tumor invasion into strap muscles, subcutaneous soft tissue, larynx, trachea, esophagus, recurrent laryngeal nerve or prevertebral fascia). Staging was performed using the American Joint Committee on Cancer (AJCC) eighth edition staging systems.

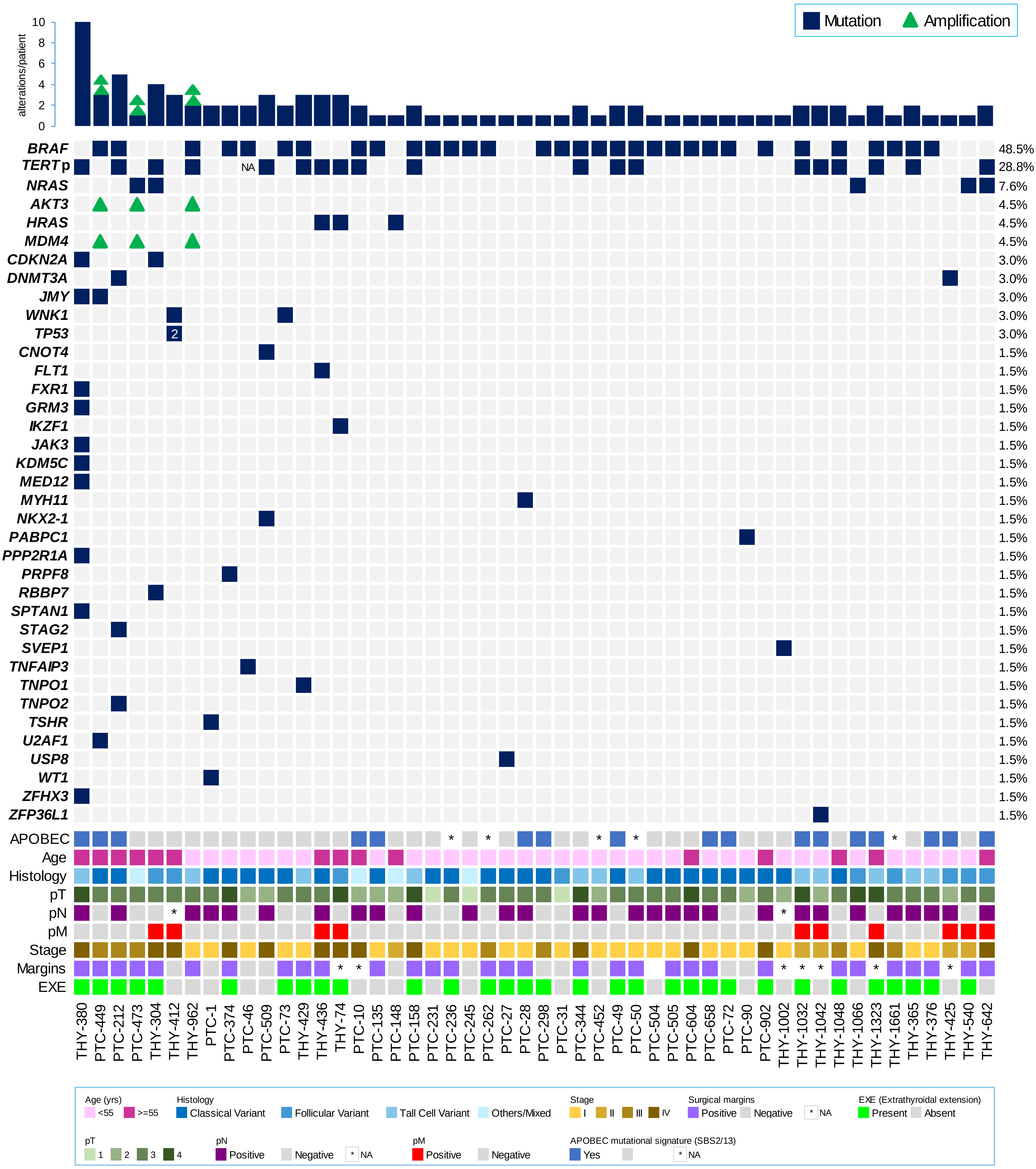

Figure S2 (A): Driver events in RAI refractory PTCs (n=50). All cancer driver mutations and copy number driver events (amplified oncogenes or deleted tumor suppressor genes) detected for each RAI refractory tumor. The top panel displays the number of driver mutations/patient identified across tumors, whereas the right panel shows the proportion of driver mutations/gene across each group. Clinical details are included for each patient. *TERT* promoter mutations (C228T and C250T) were acquired via additional Sanger Sequencing.

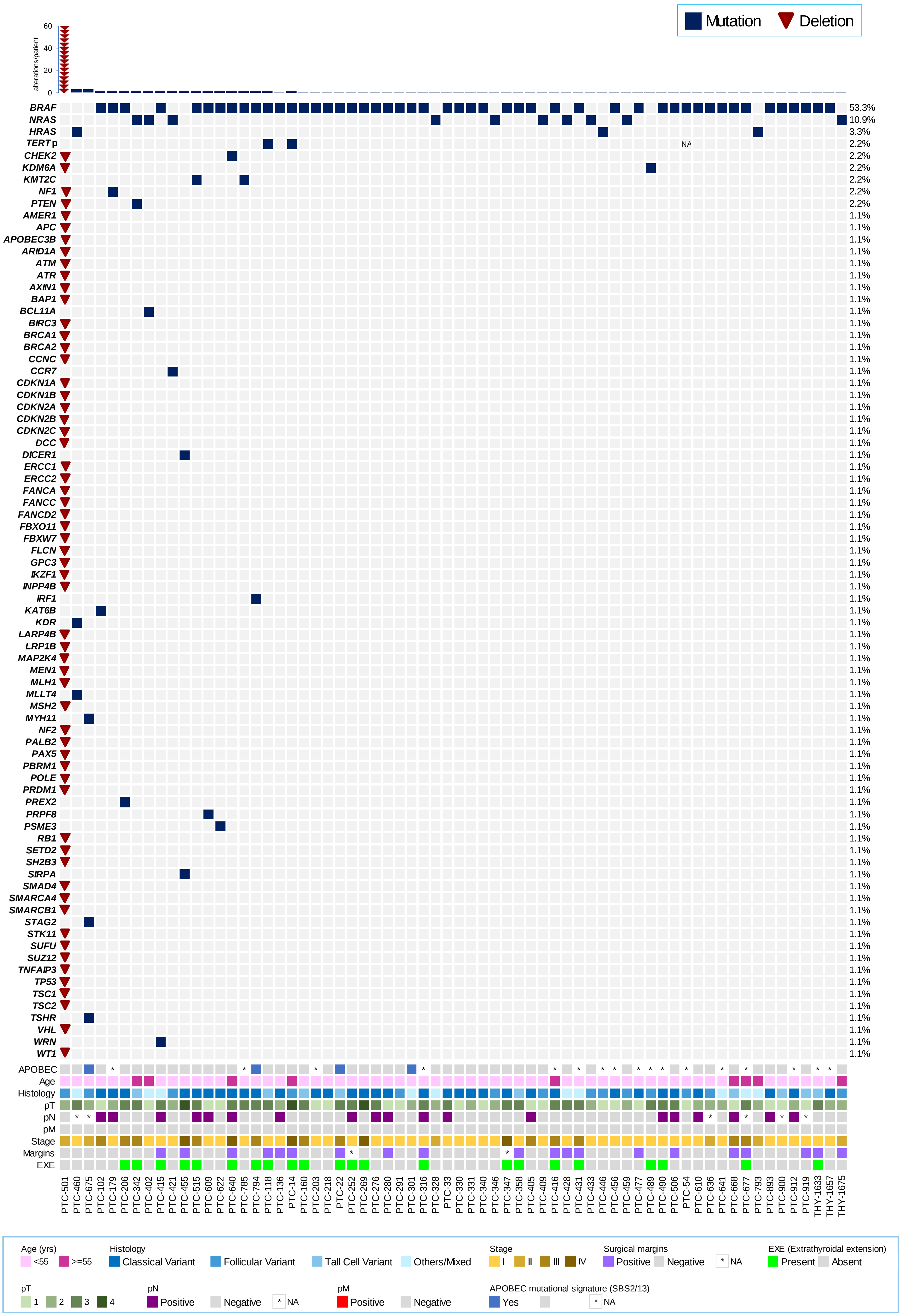

Figure S2 (B): Driver events in RAI avid PTCs (n=66). All cancer driver mutations and copy number driver events (amplified oncogenes or deleted tumor suppressor genes) detected for each RAI avid tumor. The top panel displays the number of driver mutations/patient identified across tumors, whereas the right panel shows the proportion of driver mutations/gene across each group. Clinical details are included for each patient. *TERT* promoter mutations (C228T and C250T) were acquired via additional Sanger Sequencing.

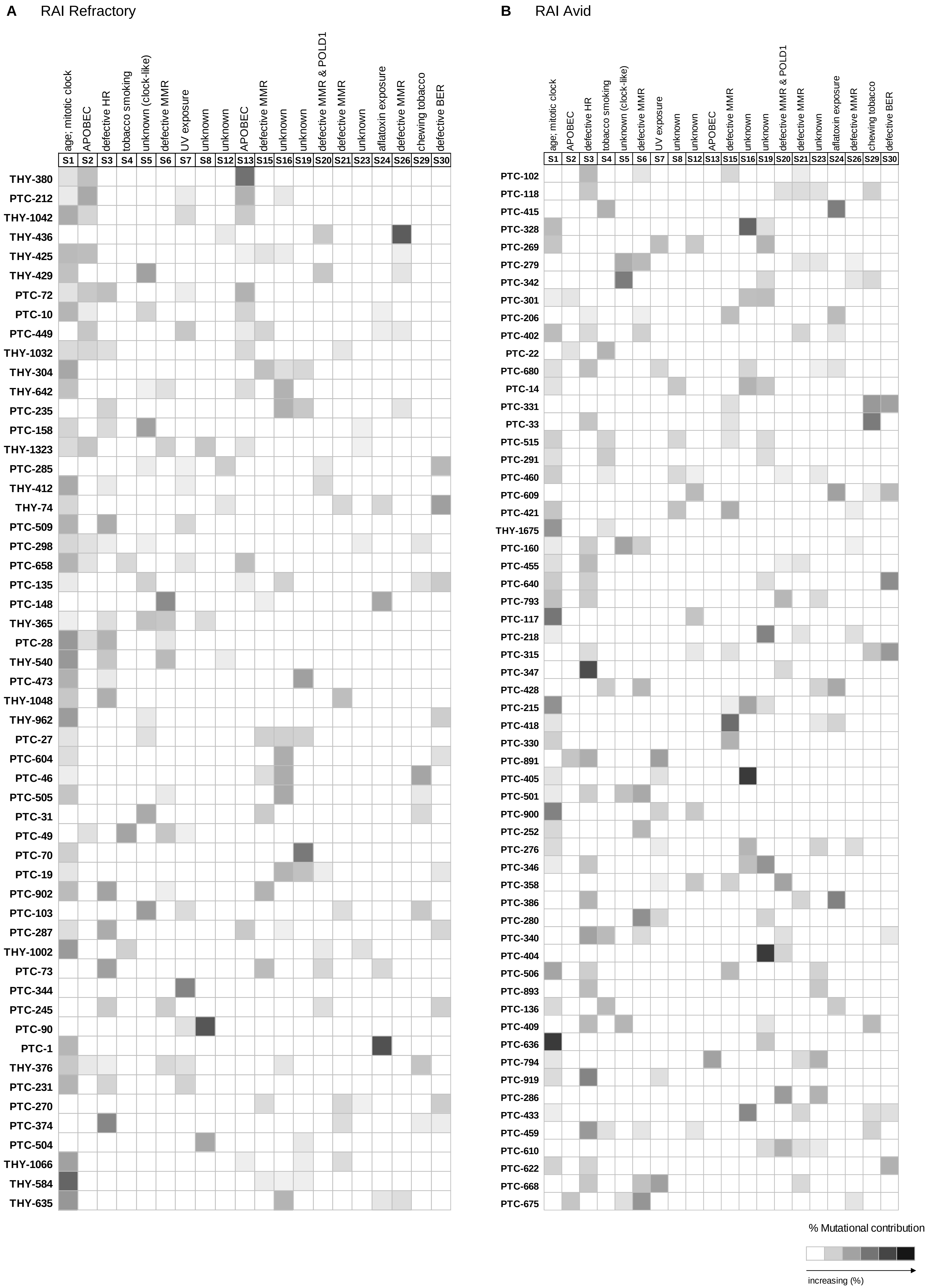

Figure S3: Twenty selected mutational signatures in individual RAI (A) refractory (n=54) and (B) avid tumors (n=59).
